# Differential Expression of Three *Cryptosporidium* Species-Specific MEDLE Proteins

**DOI:** 10.3389/fmicb.2019.01177

**Published:** 2019-05-29

**Authors:** Jiayuan Su, Chanchan Jin, Haizhen Wu, Jilan Fei, Na Li, Yaqiong Guo, Yaoyu Feng, Lihua Xiao

**Affiliations:** ^1^ State Key Laboratory of Bioreactor Engineering, School of Resources and Environmental Engineering, East China University of Science and Technology, Shanghai, China; ^2^ School of Biotechnology, East China University of Science and Technology, Shanghai, China; ^3^ Key Laboratory of Zoonosis of Ministry of Agriculture, College of Veterinary Medicine, South China Agricultural University, Guangzhou, China

**Keywords:** *Cryptosporidium parvum*, *Cryptosporidium hominis*, MEDLE family, invasion, growth

## Abstract

*Cryptosporidium parvum* and *Cryptosporidium hominis* share highly similar proteomes, with merely ~3% divergence in overall nucleotide sequences. *Cryptosporidium*-specific MEDLE family is one of the major differences in gene content between the two species. Comparative genomic analysis indicated that MEDLE family may contribute to differences in host range among *Cryptosporidium* spp. Previous studies have suggested that CpMEDLE-1 encoded by *cgd5_4580* and CpMEDLE-2 encoded by *cgd5_4590* are potentially involved in the invasion of *C. parvum*. In this study, we expressed in *Escherichia coli,* the *C. hominis*-specific member of the MEDLE protein family, ChMEDLE-1 encoded by *chro.50507,* and two *C. parvum*-specific members, CpMEDLE-3 encoded by *cgd5_4600* and CpMEDLE-5 encoded by *cgd6_5480*. Quantitative PCR, immunofluorescence staining and *in vitro* neutralization assay were conducted to assess their biologic characteristics. The expression of the *cgd5_4600* gene was high during 12–48 h of the *in vitro* culture, while the expression of *cgd6_5480* was the highest at 2 h. ChMEDLE-1 and CpMEDLE-3 proteins were mostly located in the anterior and mid-anterior region of sporozoites and merozoites, whereas CpMEDLE-5 was expressed over the entire surface of these invasive stages. Polyclonal antibodies against MEDLE proteins had different neutralization efficiency, reaching approximately 50% for ChMEDLE-1 and 60% for CpMEDLE-3, but only 20% for CpMEDLE-5. The differences in protein and gene expression and neutralizing capacity indicated the MEDLE proteins may have different roles during *Cryptosporidium* invasion and growth.

## Introduction

*Cryptosporidium* spp. are apicomplexan pathogens inhabiting the brush border of the gastrointestinal epithelium of various vertebrates, causing enterocolitis, vomiting, and watery diarrhea (Checkley et al., 2015). Hundreds of waterborne outbreaks of cryptosporidiosis have been reported around the world (Efstratiou et al., 2017). In most immune-competent individuals, the diarrhea lasts 1–2 weeks after the infection. However, immunocompromised persons such as AIDS patients may experience prolonged, life-threatening diarrhea (Chalmers and Davies, 2010). There are no effective anti-parasitic drugs for cryptosporidiosis. One of the reasons may be the lack of knowledge of the invasion process of *Cryptosporidium* spp. (Singh et al., 2015; Bhalchandra et al., 2018).

The genus *Cryptosporidium* consists of about 100 named species and genotypes that differ from each other in host specificity (Feng et al., 2018). Most of them have a narrow host range; thus, *C. hominis* mostly infects humans. A few species such as *C. parvum* have a broader range of hosts, including ruminants, rodents, and humans (Xiao, 2010; Ryan and Hijjawi, 2015). Comparative genomic analysis has shown highly similar proteomes between these two major human-pathogenic species, with merely ~3% divergence in overall nucleotide sequences (Abrahamsen et al., 2004). Differences in gene content between the two *Cryptosporidium* species are centered on two major secreted protein families, MEDLE and insulinase-like proteases (Guo et al., 2015). For the MEDLE family, named after its conserved sequence motif at the C-terminus, six subtelomeric genes are present in *C. parvum*, compared with one in *C. hominis*. It was thus suggested that MEDLE family of secreted proteins could contribute to differences in host specificity between *C. parvum* and *C. hominis* (Liu et al., 2016; Feng et al., 2017).

In previous studies, *C. parvum*-specific MEDLE-1 encoded by *cgd5_4580* and MEDLE-2 encoded by *cgd5_4590* were characterized by our group (Li et al., 2017; Fei et al., 2018). These studies supported the potential involvement of MEDLE proteins in the invasion of *C. parvum*. While MEDLE proteins share similar amino acid sequence, MEDLE-1 and MEDLE-2 are unusual members in the protein family, as their amino acid sequences end without the signal motif MEDLE at the C-terminus (Guo et al., 2015).

To further explore the functions of different MEDLE proteins, we have expressed in this study recombinant proteins of *C. hominis*-specific MEDLE-1 encoded by the *chro.50507* gene, its homolog in *C. parvum*, CpMEDLE-3 encoded by *cgd5_4600*, and the *C. parvum*-specific CpMEDLE-5 encoded by *cgd6_5480*. Through immunofluorescence microscopy, quantitative analysis of the MEDLE gene expression, and *in vitro* neutralization assays, we examined the expression profiles and potential roles of the three MEDLE proteins in host cell invasion and parasite growth.

## Materials and Methods

### *Cryptosporidium parvum* Oocyst, Host Cells, and Infection Model

*Cryptosporidium parvum* oocysts (IOWA strain) were purchased from Waterborne, Inc. (New Orleans, LA, USA), stored in antibiotics (200 U/ml penicillin, 200 μg/ml streptomycin, and 0.5 μg/ml amphotericin B) at 4°C, and used within 3 months after their harvest. *Cryptosporidium hominis* oocysts were purified from feces of naturally infected crab-eating macaques using the sucrose density gradient centrifugation method (Arrowood and Donaldson, 1996) and used within 3 weeks. The identification of *Cryptosporidium* species for isolates used in this study was made by PCR and sequence analyses of the small subunit rRNA gene (Feng et al., 2014). Prior to infection, *C. parvum* and *C. hominis* oocysts were treated on ice for 10 min with 0.5% sodium hypochlorite and washed three times with PBS. To obtain free sporozoites, sodium hypochlorite-treated oocysts were excysted in sterile PBS supplemented with 0.75% sodium taurocholate and 0.25% trypsin at 37°C for 1 h. Human ileocecal adenocarcinoma HCT-8 cells were purchased from the Chinese Academy of Sciences Shanghai Branch. Before infection experiments, HCT-8 cells were seeded into 12-well cell culture plates and cultured at 37°C in a humidified incubator containing 5% CO_2_ until they reached ~80% confluence. Each well was inoculated with sporozoites from 5 × 10^5^ oocysts in RPMI 1640 media containing 10% fetal bovine serum, 15 mM HEPES, 50 mM glucose, 10 μg/ml of bovine insulin, 35 μg/ml of ascorbic acid, 1.0 μg/ml of folic acid, 4.0 μg/ml of 4-aminobenzoic acid, 2.0 μg/ml of calcium pantothenate, 50 U/ml of penicillin G, 50 U/ml of streptomycin, and 0.25 μg/ml of amphotericin B (Mauzy et al., 2012). After incubation at 37°C for 2 h, unexcysted oocysts and free sporozoites were washed off the monolayers with sterile PBS. Fresh RPMI 1640 medium containing 2% fetal bovine serum was added to allow cells to grow for specified periods.

### Cloning of MEDLE Genes and Construction of Recombinant Plasmids

As MEDLE genes have no introns, we used *Cryptosporidium* genomic DNA to amplify the coding sequences of the target genes. The template DNA was extracted from *Cryptosporidium* oocysts by using the Qiagen DNeasy Blood & Tissue Kit (Qiagen, Hilden, Germany). Using primers incorporated with restriction sites, the *cgd5_4600* gene (XM_625308) and *cgd6_5480* (XM_625312) genes were amplified by PCR from *C. parvum* DNA while the *chro.50507* gene (XM_660938), the homolog of *cgd5_4600*, from *C. hominis* DNA. The primers used included 5′-CCGGAATTCTTTTTGGTTAAAAAAGATG-3′ (including an *EcoR*I restriction site) and 5′-CCGCTCGAGATATTTTTCCATGACCCAC-3′ (including an *Xho*I restriction site) for *chro.50507,* 5′-AAATCCATGGAAAATATAACCGATAATT-3′ (including an *Nco*I restriction site) and 5′-AAATCTCGAGTTCCAAATCTTCCATATTAA-3′ (including an *Xho*I restriction site) for *cgd5_4600,* and 5′-CGGAATTCACCGATGATTTTTTGGTTAA3′ (including an *EcoR*I restriction site) and 5′-AAATCTCGAGTTCCAAATCTTCCATATTAATA-3′ (including an *Xho*I restriction site) for *cgd6_5480*. The 50-μl PCR reaction contained 1 μl template DNA, 0.25 mM primers, 3 mM MgCl_2_, 200 μM deoxynucleotide triphosphates, 1 × GeneAmp PCR buffer (Applied Biosystems, Foster City, CA), and 1.5 U Taq polymerase (Promega, Madison, WI). The amplification was conducted on a GeneAmp 9700 (Applied Biosystems) using an initial denaturation at 94°C for 5 min; 35 cycles of amplification at 94°C for 45 s, 52°C for 45 s, and 72°C for 1 min; and one final extension at 72°C for 7 min. The PCR products were purified by using the Column PCR Product Purification Kit (Qiagen), digested with *EcoR*I*/Nco*I and *Xho*I, and ligated into double-digested vector pET28a (Novagen, Madison, WI). The recombinant plasmids were transformed into competent *E. coli* DH5α (Tiangen, Beijing, China). Positive colonies selected from solid Luria-Bertani (LB) ager containing 50 μg/ml of kanamycin were identified by PCR. They were sequenced to verify their identity and sequence accuracy.

### Expression of Recombinant MEDLE Protein in *E. coli*

To express the target proteins, recombinant plasmids were extracted from *E. coli DH5α* using the QIAGEN Plasmid Mini Kit (Qiagen) and transformed into *E. coli* BL21 (DE3) (Tiangen), which was incubated at 37°C in liquid LB medium supplemented with 50 μg/ml of kanamycin. When the OD_600_ reached 0.6, 0.5 mM isopropyl b-D-1-thiogalactopyranoside (IPTG) was introduced to induce protein expression at 25°C for 8 h. The expression level of target proteins was assessed by using sodium dodecyl sulfate polyacrylamide gel electrophoresis (SDS-PAGE) and western blot analyses. The loading buffer for the SDS-PAGE contained dl-dithiothreitol (DTT) and sodium dodecyl sulfate (SDS).

### Purification of MEDLE Proteins and Preparation of Anti-MEDLE Antibodies

The target proteins were purified using Ni-NTA beads (Novagen) and manufacturer-recommended procedures. In brief, cultured *E. coli* cells were harvested by centrifugation and lysed by sonication. The soluble fraction of the *E. coli* lysate was collected by centrifugation at 10,000 *g* for 30 min. After filtered through a 0.45 μm cellulose acetate membrane filter (Millipore, Billerica, MA), the supernatant was loaded onto Ni-NTA beads. After washing with five volumes of 20 mM imidazole buffer, target proteins were eluted with buffers containing increasing concentrations of imidazole. The purity and quantity of recombinant proteins harvested were assessed by using SDS-PAGE and a BCA kit (Yeasen, Shanghai, China). The identity of the target proteins was confirmed by using Matrix-Assisted Laser Desorption/Ionization Time of Flight Mass Spectrometry (MALDI-TOF-MS) (Murugaiyan and Roesler, 2017).

Polyclonal antibodies to all MEDLE proteins examined in this study were produced through immunizations of rabbits by GL Biochem Ltd (Shanghai, China). In the primary immunization of healthy New Zealand white rabbits, 350 μg of the purified protein mixed with Freund’s complete adjuvant was used. Boosted immunizations were conducted every 7 days for six times using 150 μg purified protein in the Freund’s incomplete adjuvant. Seven days after the last immunization, post-immune sera were harvested from the immunized animals. Pre-immune sera prior to the primary immunization were collected as negative controls. Polyclonal IgG antibodies were purified from the immune sera using protein A sepharose affinity chromatography.

### Assessment of Cross-Reactivity of Anti-MEDLE Antibodies

To assess the cross-reactivity of anti-MEDLE antibodies, the three recombinant MEDLE proteins produced in this study as well as CpMEDLE-1 and CpMEDLE-2 were employed as antigens in enzyme-linked immunosorbent assays (ELISA). They were coated onto ELISA plates at the concentration of 2 μg/ml. Antibodies against CpMEDLE-3, CpMEDLE-5 and ChMEDLE-1 and horseradish peroxidase (HRP)-conjugated goat-anti-rabbit secondary antibodies (Yeasen) were incubated in turn for the detection of reactions to the coated antigens by individual antibodies, with the pre-immune sera being used as the negative control. The reactivity of individual antibodies to each MEDLE protein was evaluated by absorbance at 450 nm. The optical density of negative control was used to compute the relative absorbance of each reaction. For example, the relative absorbance of anti-CpMEDLE-3 antibodies to CpMEDLE-5 antigen was calculated by the equation *V*_*Cp*3−*Cp*5_ = $\frac{OD_{Cp3-Cp5}}{OD_{N-Cp5}}\text{,}$ where *OD*_*Cp*3−*Cp*5_ is the difference in optical density of the reaction between anti-CpMEDLE-3 and CpMEDLE-5, and *OD*_*N–Cp*5_ is the difference in optical density of the reaction between pre-immune sera and CpMEDLE-5.

The relative absorbance of each antibody to corresponding protein was used to normalize the final data. The reactive cross-reactivity was calculated using the following formula:

Relative cross-reactivity of antibodies to antigen A against antigen B = $\frac{V_{A-B}}{V_{A-A}}$ = ODA−BODN−BODA−AODN−A.

Western blot analysis was conducted to further assess the cross-reactivity of anti-MEDLE antibodies as described above.

### Localization of MEDLE Protein Expression on Sporozoites and Developmental Stages

The reactivity of anti-MEDLE antibodies to native proteins (in crude protein extract from 1 × 10^7^ oocysts/lane) in *C. parvum* sporozoites was assessed by using western blot as described (Li et al., 2017). Polyclonal antibodies against MEDLE proteins were used as primary antibodies at the 1:4,000 dilution in PBS, while HRP-conjugated goat anti-rabbit IgG (Yeasen) as secondary antibodies at the 1:5,000 dilution in PBS, with pre-immune sera as controls. To examine MEDLE protein expression in life cycle stages, free sporozoites and intracellular parasites in HCT-8 cells on slides harvested 24 and 48 h after infection were fixed with 4% paraformaldehyde in PBS for 30 min. The fixed cells were permeabilized with 0.5% Triton X-100 in PBS for 15 min. After three washes with sterile PBS, the slides were incubated with DB Blocking Buffer (B100-40, Waterborne Inc.) at room temperature for 30 min. After another three washes with PBS, the slides were incubated with polyclonal antibodies against individual MEDLE proteins (1:8,000 diluted in PBS) for 1 h. Alexa Fluor^®^ 594-conjugated Goat Anti-rabbit IgG (Cell Signaling Technology, Danvers, MA) diluted 1:400 was used as the secondary antibodies in the immunofluorescence assay (Jakobi and Petry, 2006). Nuclei of organisms were counterstained by using 4′,6-diamidino-2-phenylindole (DAPI, Roche, Basel, Switzerland). The slides were finally mounted with No-Fade Mounting Medium (Boster, Wuhan, China), with coverslips being sealed with nail enamel. They were examined using a BX53 immunofluorescence microscope (Olympus, Tokyo, Japan) under the 400×.

### Quantitative Analysis of MEDLE Gene Expression by qPCR

The expression levels of MEDLE genes during *in vitro* development of *C. parvum* were evaluated by using qPCR. Similar work was not conducted on *C. hominis* because of the unavailability of large numbers of fresh oocysts. Total RNA was isolated from *C. parvum*-infected HCT-8 cells at 0, 2, 6, 12, 24, 36, 48, and 72 h by using the RNeasy Plus Mini Kit (Qiagen). Afterward, cDNA was synthesized from 1 μg of the total RNA using the RevertAid First Strand cDNA Synthesis Kit (Thermo Fisher Scientific). The expression of individual MEDLE genes was assessed by using qPCR analysis of the cDNA, with the final expression data being normalized with data on the expression of the *C. parvum 18S* rRNA (*Cp18S* rRNA) gene (Cai et al., 2005). Each 20-μl qPCR reaction consisted of 1 × SYBR Green Supermix (TOYOBO, Osaka, Japan), 0.1 mM primers, and 1 μl cDNA. The primers used for qPCR were as follows: primers for the *Cp18S* rRNA: 5′-CTAGAGATTGGAGGTTGTTCC-3′ and 5′-CTCCACCAACTAAGAACGGC -3′ (amplicon size = 256 bp) (Mauzy et al., 2012); primers for the *cgd5_4600* gene: 5′-ATGATCCGTTCGTCGCTTAC-3′ and 5′-ACGCCGATGTTTTCTACCTG-3′ (amplicon size = 243 bp); and primers for the *cgd6_5480* gene: 5′-TTTCCATGTGCCGACTCTAA-3′ and 5′-TTTCATCATCGCATGGTTGT-3′ (amplicon size = 146 bp). The qPCR amplifications were performed on a Light Cycler 480 (Roche) for 45 cycles (95°C for 30 s, 58°C for 30 s, and 72°C for 30 s), with an initial denaturation at 95°C for 1 min. The amplification was followed by a melt curve analysis through 95°C for 1 min, ramping down to 57°C in 2° intervals, and ramping back to 95°C for 30 s. The threshold cycle (*C_T_*) values from the qPCR were used in computing the relative levels of gene expression using the ΔΔ*C_T_* approach (Livak and Schmittgen, 2001). Three biological replicates and two technical replicates were used in each experiment. The mean values in this report were obtained from three independent experiments.

### *In vitro* Neutralization of Sporozoite Invasion

The potential involvement of MEDLE proteins in host cell invasion by *C. hominis* and *C. parvum* sporozoites was examined using neutralization assays (Kuhlenschmidt et al., 2016). HCT-8 cells were infected with sporozoites of *C. hominis* (1 × 10^5^ oocysts/well) or *C. parvum* (5 × 10^5^ oocysts/well) in the presence of immune sera, anti-MEDLE polyclonal antibodies, or pre-immune sera. The sera were used at 1:200, 1:500, and 1:1,000 dilutions, while anti-MEDLE polyclonal antibodies were used at 1:4,000 dilutions. After 2-h incubation, free sporozoites were washed off the culture, which was allowed to continue for additional 24 h. Cy3-labeled polyclonal antibodies against *C. parvum* sporozoites (Waterborne, Inc.) were used to label developmental stages as described (Upton et al., 1995). The stained culture was examined under an immunofluorescence microscope as described above. Images were taken under the 200 × and analyzed using the ImageJ software^1^. The mean parasite load per field was obtained from 50 random fields from each culture. Data from two (for *C. hominis* due to the limited availability of fresh oocysts) or three (for *C. parvum*) independent experiments were compared among treatment groups using the Student’s *t* test. Data from cultures treated with pre-immune sera were used to normalize neutralization effects of immune sera.

## Results

### Production of Recombinant MEDLE Proteins in *E. coli*

**Figure 1:**
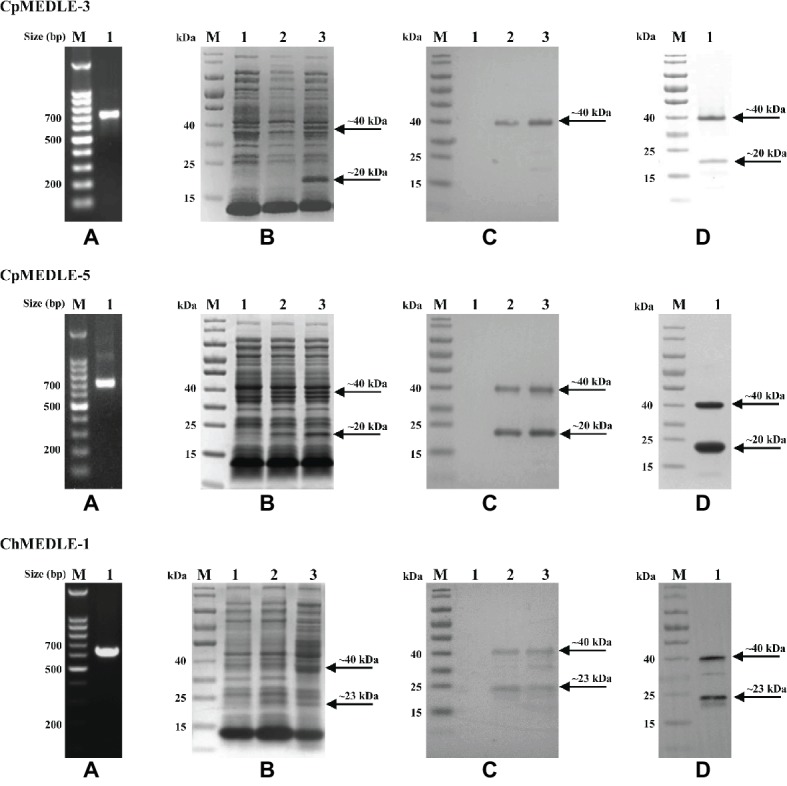
Production and purification of recombinant MEDLE proteins CpMEDLE-3 and CpMEDLE-5 from *Cryptosporidium parvum* and ChMEDLE-1 from *Cryptosporidium hominis*. **(A)** PCR amplification of the target gene in genomic DNA. Lane M: molecular markers; lane 1: PCR product. **(B)** Expression of recombinant MEDLE protein in *E. coli* BL21 (DE3) as revealed by SDS-PAGE analysis. Lane M: molecular weight markers; lane 1: lysate from bacteria culture transformed with the recombinant plasmid without IPTG induction; lane 2: lysate from similar bacteria culture induced by IPTG for 2 h; lane 3: lysate from bacteria culture induced by IPTG for 8 h, with the expected product indicated by an arrow. **(C)** Western blot analysis of the recombinant protein. Lane M: molecular weight markers; lane 1: lysate from bacteria culture transformed with recombinant plasmid without IPTG induction; lane 2: supernatant from IPTG-induced bacterial culture; lane 3: cell lysate from IPTG-induced bacterial culture. **(D)** Purification of recombinant proteins. Lane M: molecular weight markers; lane 1: purified recombinant proteins from Ni-NTA affinity chromatography.

We successfully cloned the three MEDLE genes into the pET28a vector (Figure 1A). The recombinant plasmids were sequenced to verify the identity and accuracy of DNA sequences. All three recombinant MEDLE proteins were expressed successfully at the predicted size of ~20 kDa (Figure 1B), although in western blot analysis another band of ~40 kDa was seen in products from all three recombinant plasmids (Figure 1C). Results of MALDI-TOF-MS analysis confirmed their identity, with the larger band possibly representing the dimers of the MEDLE proteins. Pure recombinant MEDLE proteins were obtained using the Ni-NTA purification method through the His-tag incorporated (Figure 1D).

### Cross-Reactivity of Antibodies to MEDLE Family Proteins

**Figure 2:**
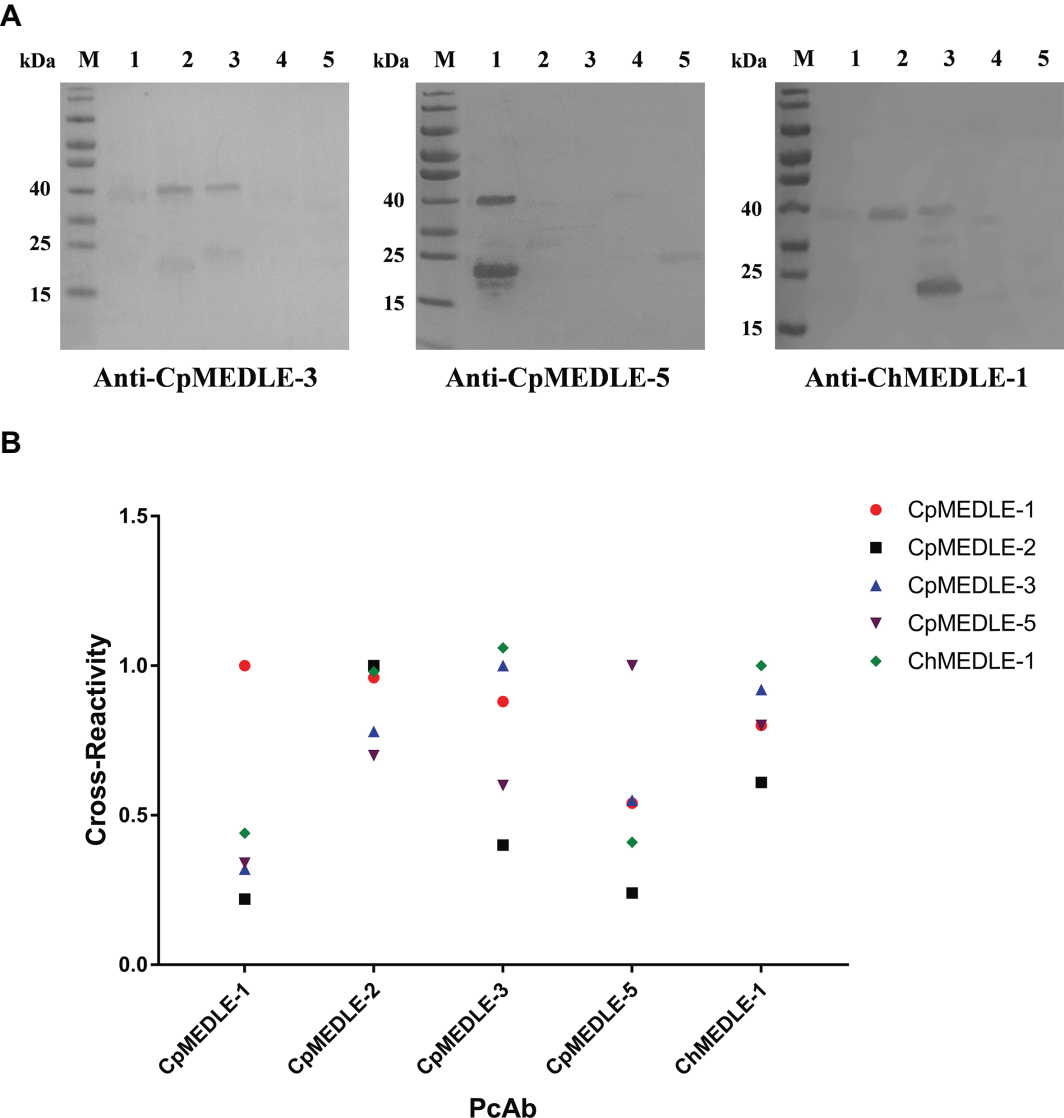
Cross-reactivity of polyclonal antibodies (PcAb) among MEDLE proteins. **(A)** Western blot analysis of cross-reactivity among MEDLE proteins, using 1 μg of CpMEDLE-5 (lane 1), CpMEDLE-3 (lane 2), ChMEDLE-1 (lane 3), CpMEDLE-1 (lane 4) and CpMEDLE-2 (lane 5) and PcAb to CpMEDLE-3 (left panel), CpMEDLE-5 (middle panel) and ChMEDLE-1 (right panel). **(B)** ELISA analysis of polyclonal antibodies to each MEDLE protein, with data being normalized using the relative absorbance of each antibody to the corresponding protein.

In western blot analysis, anti-CpMEDLE-3 antibodies showed significant cross-reactivity to ChMEDLE-1, and mainly reacted with the ~40 kDa dimers of CpMEDLE-3 and ChMEDLE-1 (Figure 2A). Anti-ChMEDLE-1 antibodies reacted with ~20 kDa monomers of ChMEDLE-1 and the ~40 kDa dimers of CpMEDLE-3, with weak reactivity to other MEDLE proteins. In contrast, anti-CpMEDLE-5 antibodies showed minimum cross-reactivity to other MEDLE proteins. Similar observations were made in ELISA analysis of the cross-reactivity, with CpMEDLE-3 and ChMEDLE-1 being recognized by ChMEDLE-1 and CpMEDLE-3 antibodies, respectively, and minimum reactivity of CpMEDLE-1 or CpMEDLE-5 antibodies to other MEDLE proteins (Figure 2B). Thus, anti-ChMEDLE-1 antibodies were expected to react with *C. parvum* sporozoites due to its cross-reactivity to CpMEDLE-3.

### Expression of MEDLE Proteins in *C. parvum* Sporozoites

**Figure 3:**
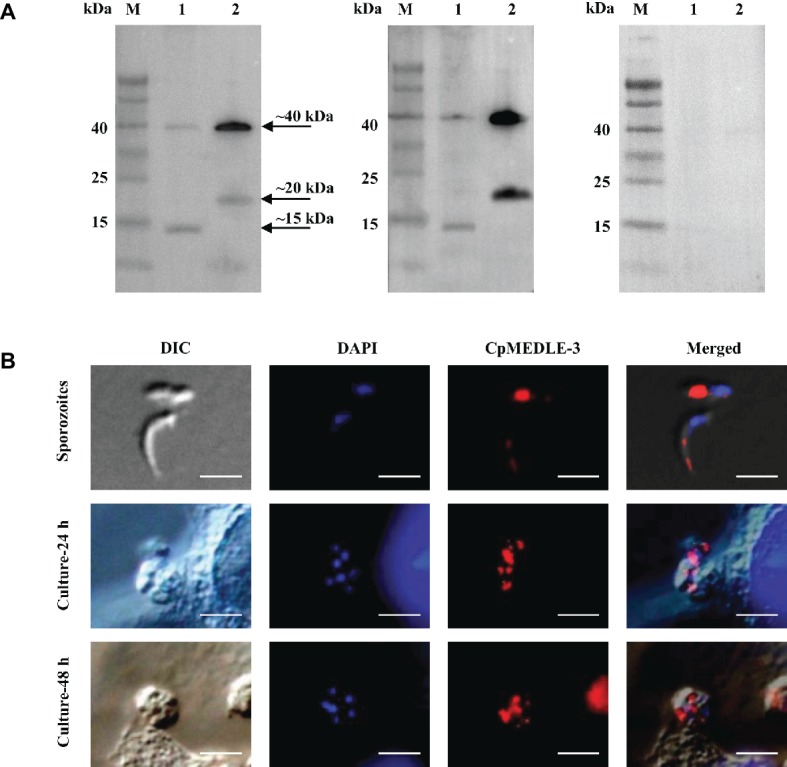
Expression of CpMEDLE-3 in sporozoites and developmental stages of *Cryptosporidium parvum*. **(A)** Western blots analysis of native protein from *C. parvum* sporozoites for CpMEDLE-3, using polyclonal antibodies (left panel), post-immune sera (middle panel) and pre-immune sera (right panel). Lane M: molecular weight markers; lane 1: crude protein extracted from sporozoites; lane 2: purified CpMEDLE-3 protein. **(B)** Expression of CpMEDLE-3 on *C. parvum* sporozoites (top panel) and intracellular developmental stages in HCT-8 cell cultures at 24 h (middle panel) and 48 h (bottom panel). The images were taken under differential interference contrast (DIC), with nucleus counter-stained with 4′,6-diamidino-2-phenylindole (DAPI), parasites stained by immunofluorescence with Alexa 594-labled CpMEDLE-3 (CpMEDLE-3), and superimposition of the three images (Merged). Bars = 5 μm.

**Figure 4:**
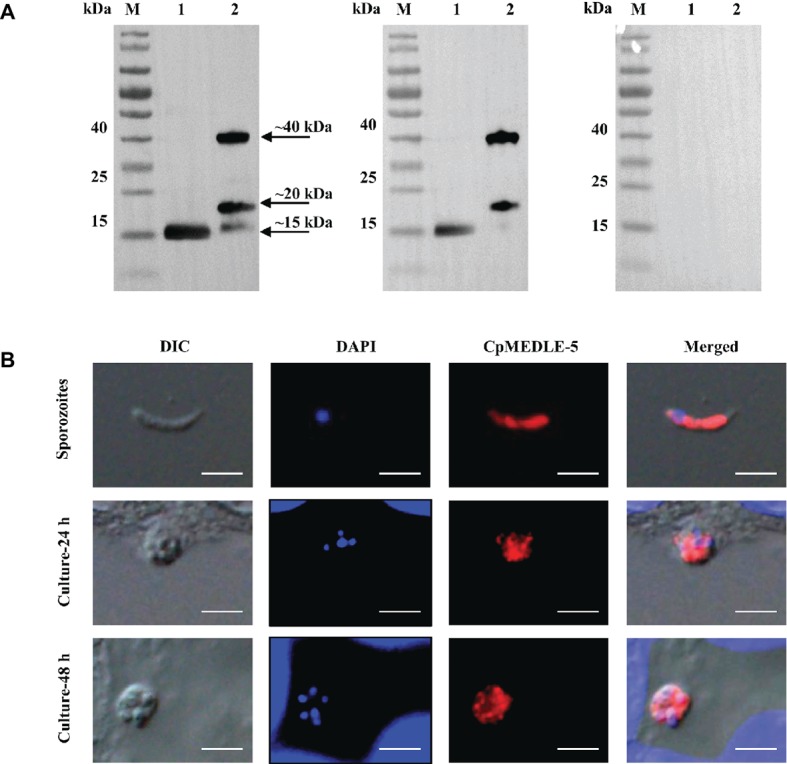
Expression of CpMEDLE-5 in sporozoites and developmental stages of *Cryptosporidium parvum*. **(A)** Western blots analysis of native protein from *C. parvum* sporozoites for CpMEDLE-5, using polyclonal antibodies (left panel), post-immune sera (middle panel) and pre-immune sera (right panel). Lane M: molecular weight markers; lane 1: crude protein extracted from sporozoites; lane 2: purified CpMEDLE-5 protein. **(B)** Expression of CpMEDLE-5 on *C. parvum* sporozoites (top panel) and intracellular developmental stages in HCT-8 cell cultures at 24 h (middle panel) and 48 h (bottom panel). The images were taken under differential interference contrast (DIC), with nucleus counter-stained with 4′,6-diamidino-2-phenylindole (DAPI), parasites stained by immunofluorescence with Alexa 594-labled CpMEDLE-5 (CpMEDLE-5), and superimposition of the three images (Merged). Bars = 5 μm.

**Figure 5:**
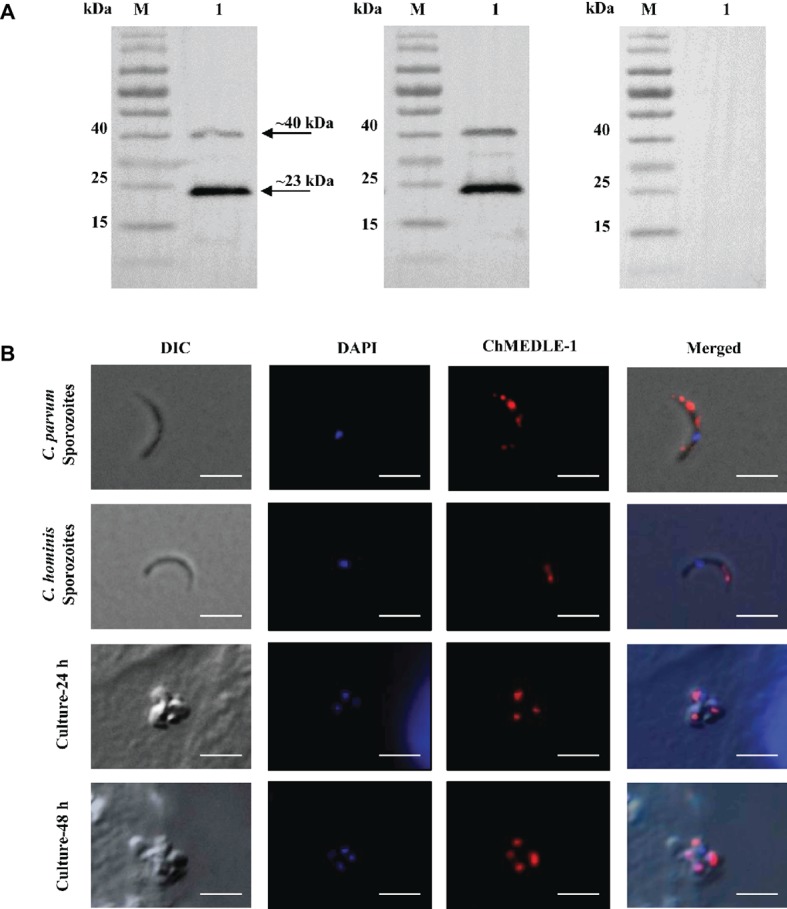
Expression of ChMEDLE-1. **(A)** Western blots analysis of ChMEDLE-1 recombinant protein, using polyclonal antibodies (left panel), post-immune sera (middle panel) and pre-immune sera (right panel). Lane M: molecular weight markers; lane 1: purified ChMEDLE-1 protein. **(B)** Expression of ChMEDLE-1 on *C. parvum* sporozoites (top panel), *C. hominis* sporozoites (the second panel) and intracellular developmental stages of *C. hominis* in HCT-8 cell cultures at 24 h (the third panel) and 48 h (bottom panel). The images were taken under differential interference contrast (DIC), with nucleus counter-stained with 4′,6-diamidino-2-phenylindole (DAPI), parasites stained by immunofluorescence with Alexa 594-labled ChMEDLE-1 (ChMEDLE-1), and superimposition of the three images (Merged). Bars = 5 μm.

All three anti-MEDLE antibodies or post-immune sera recognized their corresponding MEDLE proteins, while pre-immune sera did not react with sporozoites proteins and recombinant proteins in western blot assay (Figures 3A, 4A, 5A). The monomers and dimers of the three recombinant MEDLE proteins were all detected by using anti-MEDLE antibodies or post-immune sera. Native MEDLE proteins in *C. parvum* sporozoite extracts were recognized by the anti-CpMEDLE-3 antibodies and immune sera, generating two bands of ~15 and ~40 kDa (Figure 3A). A band of ~15 kDa protein in sporozoite extracts was recognized by CpMEDLE-5 antibodies and immune sera, but no dimerization of CpMEDLE-5 was observed (Figure 4A). Western blot analysis of native proteins was not conducted for *C. hominis* due to limited oocyst availability.

### Localization of MEDLE Proteins on Sporozoites and Developmental Stages

The expression location of MEDLE proteins in sporozoites and developmental stages was examined by immunofluorescence microscopy. CpMEDLE-3 antibodies reacted mostly with the anterior and mid-anterior regions of *C. parvum* sporozoites (Figure 3B), while CpMEDLE-5 antibodies reacted with the entire surface of *C. parvum* sporozoites (Figure 4B). The expression of CpMEDLE-3 on *C. parvum* sporozoites was also confirmed by using anti-ChMEDLE-1 antibodies (Figure 5B). ChMEDLE-1 was expressed mainly in the mid-anterior region of *C. hominis* sporozoites (Figure 5B). No reactivity to *C. parvum* or *C. hominis* sporozoites was detected using pre-immune sera (data not shown).

In assessment of MEDLE expression in intracellular stages at 24 and 48 h of cultures, CpMEDLE-3 and ChMEDLE-1 antibodies reacted with only part of the merozoites opposite to the nucleus (Figures 3B, 5B). In contrast, the reactivity of CpMEDLE-5 antibodies dispersed throughout the meronts, including the merozoites within them (Figure 4B). Antibodies against all three MEDLE proteins did not recognize the parasitophorous vacuole.

### Expression of MEDLE Gene in *C. parvum* Culture

**Figure 6:**
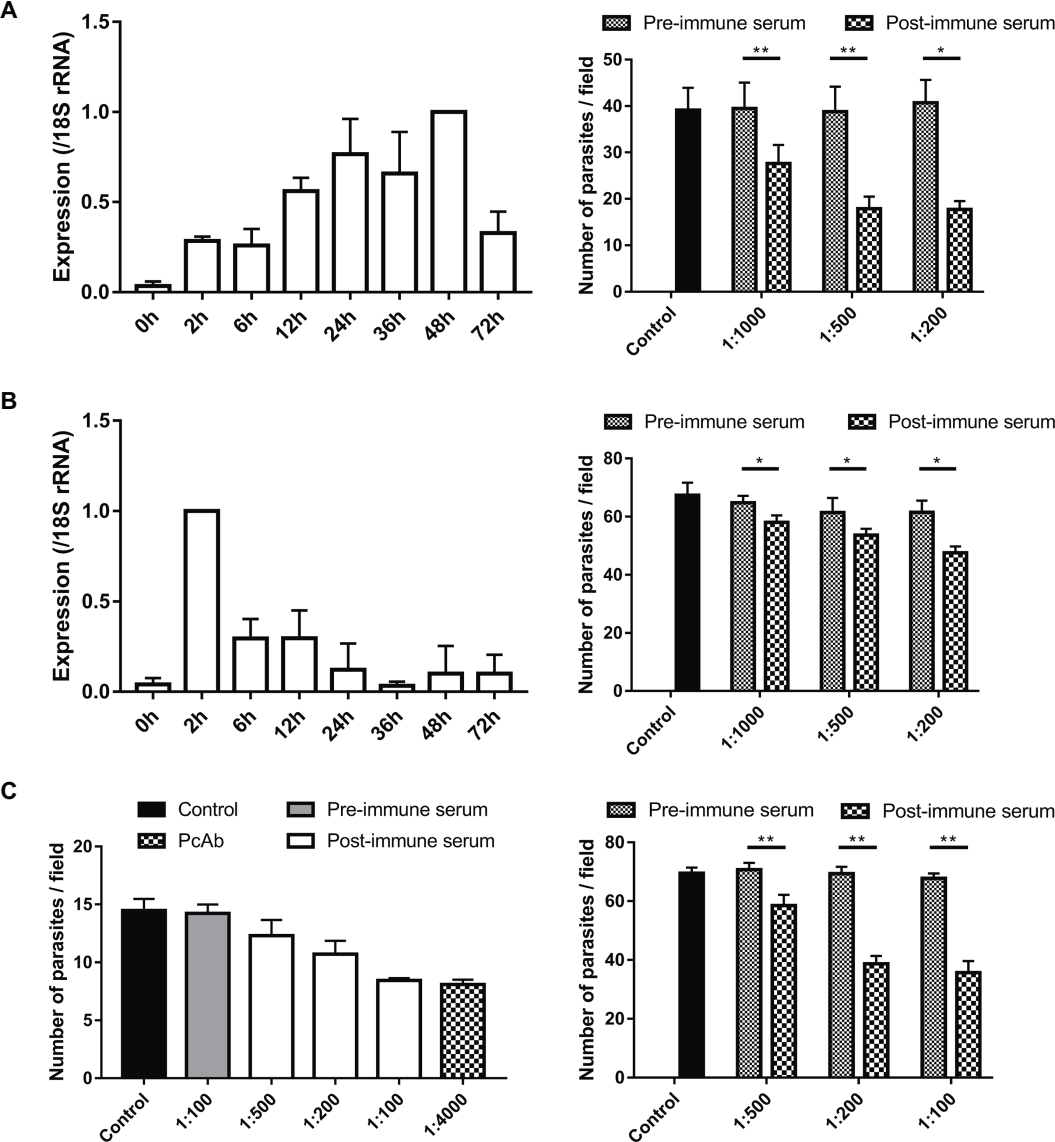
Assessment of biological functions of MEDLE proteins. **(A)** Expression levels of the *cgd5_4600* gene in developmental stages of *C. parvum* (left panel) and neutralization efficiency of immune sera against CpMEDLE-3 on *C. parvum* invasion (right panel). The expression level of the *cgd5_4600* gene in *C. parvum* culture was determined by qPCR at various time points, with data being normalized with data from the expression of the *Cp18S* rRNA. Neutralization efficiency of post-immune sera against CpMEDLE-3 on *C. parvum* invasion was measured in HCT-8 cell culture. Data presented are mean ± SD from three independent experiments for both the expression and neutralization studies. **(B)** Expression levels of the *cgd6_5480* gene in developmental stages of *C. parvum* (left panel) and neutralization efficiency of immune sera against CpMEDLE-5 on *C. parvum* invasion (right panel). The expression level of the *cgd6_5480* gene in *C. parvum* culture was determined by qPCR at various time points, with data being normalized with data from the expression of the *Cp18S* rRNA. Neutralization efficiency of immune sera against CpMEDLE-5 on *C. parvum* invasion was measured in HCT-8 cell culture. Data presented are mean ± SD from three independent experiments for both the expression and neutralization studies. **(C)** Neutralization efficiency of polyclonal antibodies (PcAb) and immune sera against ChMEDLE-1 on *C. hominis* (left panel) and *C. parvum* (right panel) invasion. Cultures were treated with PcAb, immune sera, or pre-immune sera. Data presented are mean ± SD from two (for *C. hominis*) or three (for *C. parvum*) independent experiments. The statistical significance of differences between treatment groups is indicated for neutralization studies of all three MEDLE proteins above the bars (*p < 0.05; **p < 0.01).

The relative expression levels of the MEDLE genes during intracellular development of *C. parvum* in cell culture during the first 72 h were assessed by qPCR in three independent experiments. The expression of the *cgd5_4600* gene increased gradually during the parasite development, with high levels of expression sustained from 24 to 48 h of culture (Figure 6A, left panel). In contrast, the expression of the *cgd6_5480* gene was highest at 2 h of culture, with a drastic reduction thereafter till 72 h (Figure 6B, left panel). The expression of the *chro.50507* gene for ChMEDLE-1 in *C. hominis* was not assessed due to the lack of sufficient fresh oocysts.

### Neutralization of Sporozoite Invasion by MEDLE Antibodies

Invasion neutralization assays were used to assess the effect of anti-MEDLE antibodies on the infection of HCH-8 cells with *C. parvum* (in three independent experiments) or *C. hominis* (in two independent experiments). Compared with control cultures, a significant reduction in *C. parvum* load was seen when sporozoites were pre-incubated with antisera from CpMEDLE-3 immunized rabbits (Figure 6A, right panel). The inhibition rate was 30.1% [39.5 ± 5.6 and 27.6 ± 4.0 per 200 × field for pre- and post-immune sera, respectively; *t*_(2)_ = 12.931, *p* = 0.006] at 1:1,000 dilution, 53.9% [38.8 ± 5.4 and 17.9 ± 2.6 per 200 × field for pre- and post-immune sera, respectively; *t*_(2)_ = 12.144, *p* = 0.007] at 1:500 dilution, and 56.5% [40.7 ± 5.0 and 17.7 ± 1.8 per 200 × field for pre- and post-immune sera, respectively; *t*_(2)_ = 8.549, *p* = 0.013] at 1:200 dilution. The parasite load in cultures with no addition of rabbit sera was 39.1 ± 4.8 per 200 × field. In contrast, antisera generated from CpMEDLE-5 immunization did not have any similar effects on *C. parvum* invasion (Figure 6B, right panel), with only 10.3% [64.7 ± 2.4 and 58.1 ± 2.4 per 200 × field for pre- and post-immune sera, respectively; *t*_(2)_ = 7.551, *p* = 0.017], 12.8% [61.5 ± 4.9 and 53.7 ± 2.1 per 200 × field for pre- and post-immune sera, respectively; *t*_(2)_ = 4.594, *p* = 0.044], and 22.6% [61.5 ± 4.0 and 47.6 ± 2.2 per 200 × field for pre- and post-immune sera, respectively; *t*_(2)_ = 4.329, *p* = 0.049] reductions at 1:1,000, 1:500, and 1:200 dilutions, respectively.

In neutralization assays conducted with antisera from ChMEDLE-1 immunization, when HCT-8 cells were infected with *C. hominis* (Figure 6C, left panel), the highest inhibition rate reached 40.6% at 1:100 dilution of immune sera [14.2 ± 0.8 and 8.5 ± 0.2 per 200 × field for pre- and post-immune sera, respectively; *t*_(1)_ = 14.073, *p* = 0.045]. A similar inhibition of *C. hominis* invasion was achieved with the addition of purified anti-ChMEDLE-1 antibodies at 1:4,000 dilution: 43.1% [14.5 ± 1.0 and 8.1 ± 0.4 per 200 × field for no addition culture and polyclonal antibodies, respectively; *t*_(1)_ = 15.190, *p* = 0.042]. When HCT-8 cell culture was inoculated with *C. parvum* sporozoites treated with antisera against ChMEDLE-1 (Figure 6C, right panel), the inhibition rate was 17.3% [70.7 ± 2.3 and 58.5 ± 3.7 per 200 × field for pre- and post-immune sera, respectively; *t*_(2)_ = 15.408, *p* = 0.004], 44.2% [69.4 ± 2.4 and 38.7 ± 2.7 per 200 × field for pre- and post-immune sera, respectively; *t*_(2)_ = 16.424, *p* = 0.004], and 47.3% [67.8 ± 1.6 and 35.7 ± 4.0 per 200 × field for pre- and post-immune sera, respectively; *t*_(2)_ = 14.396, *p* = 0.005] at 1:500, 1:200, and 1:100 dilutions, respectively.

## Discussion

In this study, we provided evidence for the hypothesis that members of MEDLEs may have different expression and roles in the invasion and growth of *C. parvum* and *C. hominis*, although the precise functions of these proteins remain unclear. Most proteins in the *Cryptosporidium* proteome are encoded by single-copy genes. The MEDLE proteins are among the few *Cryptosporidium*-specific proteins encoded by gene families. The multi-copy nature of these proteins signals their biological importance. As members of the protein family have similar sequences, they could serve as a strategy for the parasite to diversify its interactions with host cells, especially during the invasion process, such as increased range of susceptible hosts or host cells (reduced host specificity or tissue tropism). Alternatively, these genes have regulated expression, producing different members exerting their functions at specific life cycle stages. Both scenarios appear likely for *C. parvum*, which has a broader host range *C. hominis*. The latter, however, has only one copy of the MEDLE genes, but appears to have the invasion process and growth pattern similar to *C. parvum*.

Despite the significant sequence similarity among MEDLE proteins, they have different spectrum of cross-reactivity of antibodies raised against individual recombinant proteins. Some of the antibodies are more specific, such as those raised against CpMEDLE-1 and CpMEDLE-5, which showed minimum reactivity to other MEDLE proteins. Others, especially those against CpMEDLE-2, cross-react mostly with other MEDLE proteins. ChMEDLE-1 and CpMEDLE-3 have very similar spectrum of antibody cross-reactivity, probably due to their high amino acid sequence identity (67%). In the native protein expression, CpMEDLE-3 is in the forms of monomer (~15 kDa) and dimer (~40 kDa), while CpMEDLE-5 forms only monomer (~15 kDa). In previous studies, CpMEDLE-2 did not form dimers either, while CpMEDLE-1 reacted with fragments of ~32, ~36, and ~50 kDa in *C. parvum* sporozoite extracts (Li et al., 2017; Fei et al., 2018). These differences in dimer formation among MEDLE proteins in sporozoites support the conclusion on their differential roles. It would be important to determine whether dimerization is critical to the functions or activities of MEDLE proteins.

The functional diversity of MEDLE proteins is supported by their expression patterns in sporozoites and developmental stages. All MEDLE proteins are expressed on both sporozoites and merozoites, the invasive stages of *Cryptosporidium* spp. CpMEDLE-3 is expressed in the anterior and mid-anterior regions of *C. parvum* sporozoites, as indicated by immunofluorescence analyses using both anti-CpMEDLE-3 and anti-ChMEDLE-1 antibodies. In contrast, CpMEDLE-1 and ChMEDLE-1 are similarly expressed, while CpMEDLE-2 and CpMEDLE-5 are expressed over almost the entire *C. parvum* sporozoites (Li et al., 2017; Fei et al., 2018). Previous studies reported that most invasion-associated proteins were located in anterior or mid-anterior regions of sporozoites (Singh et al., 2015). Thus, among the three proteins analyzed in the present study, CpMEDLE-3 and ChMEDLE-1 are more likely to play some roles in the invasion of *Cryptosporidium*. As MEDLE proteins are secretory proteins, these differences in expression patterns could be due to their different subcellular locations. In particular, the dotted location of ChMEDLE-1 on sporozoites and merozoites suggests that it could be a dense granule protein. As the homolog of ChMEDLE-1 in *C. parvum*, CpMEDLE-3 as expectedly has a similar expression pattern on sporozoites and merozoites; thus, could also be a dense granule protein, while CpMEDLE-2 and CpMEDLE-5 could be cytoplasmic proteins. Dense granule proteins are known to play critical roles in the invasion and virulence of other apicomplexan parasites such as *Toxoplasma gondii* (Gubbels and Duraisingh, 2012).

The gene expression profile of MEDLE proteins further illustrates their differential roles in invasion and growth of *C. parvum*. The genes encoding these proteins have peak expressions at different time during parasite invasion and growth; genes encoding CpMEDLE-1 and CpMEDLE-5 have the highest expression at 2 h after invasion, the gene for CpMEDLE-2 peaks at 48 h, while the one for CpMEDLE-3 has high expression after 12 h (Li et al., 2017; Fei et al., 2018). The differences in protein subcellular locations and gene transcription patterns agree with the theory that MEDLE proteins play different roles during parasite invasion and growth of *C. parvum*. The lack of some of the MEDLE proteins in *C. hominis* could be responsible for some of the biological differences between the two *Cryptosporidium* species, including host specificity.

In neutralization examination, polyclonal antibodies against individual MEDLE proteins have different abilities in inhibiting parasite invasion of HCT-8 cells. For instance, CpMEDLE-5 antibodies inhibited the invasion efficiency only marginally, while antibodies against CpMEDLE-3 reduced the invasion by nearly 60%. The inhibition rate obtained in this study is similar to rates reported in previous studies of several invasion-related proteins. For instance, antisera against gp40, a protein which was involved in *C. parvum*-host cell interactions, inhibited *C. parvum* infection by 68% (Cevallos et al., 2000). Polyclonal antibodies to EF-1*α*, which forms an essential component of the invasion apparatus of the parasite, blocked *C. parvum* infection of HCT-8 cells by 80.9% in a neutralization assay (Matsubayashi et al., 2013). Differences in the neutralization efficiency of antibodies could be due to differences in the roles of various MEDLE proteins during invasion and growth. Among the five MEDLE proteins of *C. parvum* and *C. hominis* examined thus far, CpMEDLE-3 and its homolog ChMEDLE-1, both apparently dense granule proteins, have generated the highest inhibition of *Cryptosporidium* invasion, indicating their important roles in the invasion of *C. parvum* and *C. hominis*. Interestingly, ChMEDLE-1 is the only MEDLE member in *C. hominis*. Antibodies against ChMEDLE-1 showed significant ability to neutralize the infection of HCT-8 by both *C. hominis* and *C. parvum*. As ChMEDLE-1 is the homolog of CpMEDLE-3 in *C. parvum*, the neutralization of *C. parvum* infection by of antibodies against ChMEDLE-1 is expected. As there was minimal cross-reactivity in antibodies among other MEDLE proteins of *C. parvum*, results of the neutralization studies agree with the suggestion that MEDLE proteins may contribute to the host specificity of *Cryptosporidium* spp.

Although we have obtained some preliminary evidence to support the suggestion that MEDLE proteins are differentially expressed and play different roles in the *Cryptosporidium* lifecycle, more biologic and immunohistological studies, including the use of genetic manipulation of the pathogen by CRISPR/Cas9, are needed to better understand the mechanism of MEDLE proteins during invasion of various *Cryptosporidium* spp. (Beverley, 2015). Considering the significant inhibition rates offered by their antibodies, MEDLE proteins could be potential targets in the design of new drugs or vaccines against cryptosporidiosis. The elucidation of crystal structures of MEDLE proteins would be a logic move toward this goal. These studies are likely leading to improved understanding of the invasion process of *Cryptosporidium* spp. and the development of new intervention strategies.

## Data Availability

All datasets generated for this study are included in the manuscript and/or the supplementary files.

## Author Contributions

YF and LX designed the study. JS, CJ, and JF performed the experiments. JS, YF, HW, NL, YG, and LX performed the statistical analysis and interpreted the results. JS, YF, and LX developed the draft manuscript. All authors contributed to manuscript revisions and approved the final version for publication.

### Conflict of Interest Statement

The authors declare that the research was conducted in the absence of any commercial or financial relationships that could be construed as a potential conflict of interest.

## Footnotes

^1^https://imagej.nih.gov/ij/

## Abbreviations

DAPI: 4′,6-Diamidino-2-phenylindole
DIC: Differential interference contrast
ELISA: Enzyme-linked immunosorbent assay
HRP: Horseradish peroxidase
IPTG: Isopropyl b-D-1-thiogalactopyranoside
MALDI-TOF-MS: Matrix-assisted laser desorption/ionization time of flight mass spectrometry
PBS: Phosphatebuffered saline
PcAb: Polyclonal antibodies
SDS-PAGE: Sodium dodecyl sulfate polyacrylamide gel electrophoresis.
